# Supplementary material for: A Comprehensive Overview of Intraoperative Complications during Retzius-Sparing Robot-Assisted Radical Prostatectomy: Single Series from High-Volume Center
Source: Cancers (Basel). 2024 Mar 31;16(7):1385. doi: 10.3390/cancers16071385 (PMC11010834; doi:10.3390/cancers16071385)
Supplement: Supplementary file 1 [file cancers-16-01385-s001.zip › cancers-2891147-supplementary.pdf]

**Supplementary Table S1. Intraoperative Complications Assessment and Reporting with Universal Standards (ICARUS) criteria for reporting adverse events during surgical procedures.**		
**V or X**	**Criteria**	**Explanation**
V	1. In a study reporting perioperative outcomes, iAEs should be reported as one of the outcomes of interest	In the current study intraoperative adverse events were the primary end points
V	2. iAEs and the definition of each specific iAE should be reported or referenced	Definition and description of each intraoperative adverse event were reported in a dedicated table
V	3. Each iAE should be reported using one of the proposed classification systems (ClassIntra, EAU, iAE severity classification scheme, or modified Satava), with a preference for schemes that are validated	Intraoperative adverse events were categorized according to the Intraoperative Adverse Incident Classification (EAUiaiC) proposed by the European Association of Urology (EAU) ad hoc Complications Guidelines Panel
V	4. Each iAE should be reported separately by grade	Each intraoperative adverse event was reported separately with the corresponding EAUiaiC grade
V	5. iAEs related to anesthesiology, surgery, and equipment malfunction should be reported separately	In the current study each type of intraoperative adverse event was reported separately, specifying its surgical, anesthesiologic or equipment-related nature
V	6. The number of iAEs and the number of patients with iAEs should be reported separately	Number of intraoperative adverse events and patients were reported separately
X	7. When appropriate, pre-existing medical conditions, atypical anatomical variants, and malfunctioning surgical instruments associated with iAEs should be reported	No specific pre-existing medical conditions or atypical anatomical variants were available
V	8. If an iAE requires conversion during surgery, both the iAE that caused the conversion and the action undertaken should be reported	In the current study every intraoperative adverse event requiring conversion was reported
V	9. iAEs should be reported, specifying the surgical step that was associated with or affected by the iAEs	In the current study the surgical step associated with the intraoperative adverse event was reported
V	10. The timing of iAE assessment should be reported as follows:<br>If an iAE is recognized during the surgical procedure, hold a debriefing after the surgical procedure. If an iAE is not recognized during the surgical procedure, report the point at which the iAE became apparent in the postoperative course	No intraoperative adverse events were recognized after surgery
V	11. The management of iAEs should be reported	The management of each intraoperative adverse event was not reported in a dedicated table
X	12. Report the clinical consequences of a given iAE in the postoperative course as follow:<br>a) Without postoperative sequelae<br>b) With nonpermanent postoperative sequelae<br>c) With a permanent postoperative sequela<br>d) Requiring reoperation<br>e) Postoperative death	Clinical consequences of the intraoperative adverse events were not available
X	13. Report changes to the clinical course that were associated with any iAEs	The changes to the clinical course associated with intraoperative adverse events were not systemically reported

Description of the complication and EAUiaiC grade		n, (%)	During RARP/ePNLD
Anaesthesiologic ICs, n = 2 (5%)	Desaturation during pneumoperitoneum induction requiring open conversion<br>Grade 2	1 (2.5)	
	Hemodynamic instability during ePLND, only one side performed<br>Grade 4b	1 (2.5)	
Access and trocar placement ICs, n = 5 (12.5%)	Epigastric Artery Injury<br>Grade 1	5 (12.5)	RARP
Injury of intra-abdominal organs, n = 15 (37.5%)	Bladder injuries managed with immediate repair<br>Grade 1	5 (12.5)	RARP
	Sigma Injuries managed with immediate repair<br>Grade 1	4 (10)	RARP
	Small Bowel injurie requiring suture<br>Grade 1	5 (12.5)	RARP
	Severe Small Bowel injury requiring resection and anastomosis<br>Grade 2	1 (2.5)	RARP
Vascular injuries, n = 9 (22.5%)	Minor Internal Iliac Artery injuries<br>Grade 1	3 (7.5)	ePNLD
	Major Internal Iliac Artery injury<br>Grade 3	2 (5)	ePNLD
	Gluteal Vein injuries<br>Grade 1	2 (5)	ePNLD
	Iliac Vein injuries<br>Grade 1	2 (5)	ePNLD
Nerve, n = 3 (7.5%)	Obturatory Nerve injury<br>Grade 2	3 (7.5)	ePNLD
Ureteric injuries n = 4 (10%)	Ureteral injury with suture and stenting<br>Grade 2	2 (5)	ePNLD
	Ureteral injury with anastomosis/reimplantation<br>Grade 4a	2 (5)	1 RARP<br>1ePNLD
Others n = 2 (5%)	Needle loss<br>Grade 1	1 (2.5)	RARP
	Robot malfunctioning<br>Grade 4b	1 (2.5)	ePNLD
